# Impact of regional and national milk allergy in primary care guidelines and training program on recognition and treatment of cow’s milk allergy

**DOI:** 10.1186/2045-7022-5-S3-P157

**Published:** 2015-03-30

**Authors:** Lucas Wauters, Trevor Brown, Carina Venter, Adam T Fox, Joanne Walsh, Breege Brogan, Jenny Hughes, Robert Dziubak, Neil Shah

**Affiliations:** 1 KU Leuven, Translational Research Centre for Gastrointestinal Disease (TARGID), Leuven, Belgium; 2 The Children's Allergy Service, The Ulster Hospital, Northern Ireland, United Kingdom; 3 The David Hide Asthma and Allergy Research Centre, Newport, Isle of Wight, United Kingdom; 4 School of Health Sciences and Social Work, University of Portsmouth, Portsmouth, United Kingdom; 5 MRC and Asthma UK Centre in Allergic Mechanisms of Asthma, King's College London, London, United Kingdom; 6 Department of Paediatric Allergy, Guy's and St Thomas' Hospitals, NHS Foundation Trust, London, United Kingdom; 7 Castle Partnership, Gurney Surgery, Norwich, United Kingdom; 8 Health and Social Care Board, Gransha Park, Londonderry, United Kingdom; 9 Antrim Hospital, Northern HSC Trust, Northern Ireland, United Kingdom; 10 Department of Gastroenterology, Great Ormond Street Hospital for Children NHS Foundation Trust, University College London, London, United Kingdom

## Background

Cow's milk allergy (CMA) is the commonest food allergy in UK children with many misleading presentations. In 2011, the National Institute for Health and Care Excellence (NICE) provided primary care guidelines on food allergy. In Northern Ireland (NI), a regional expert group was convened in 2012 to develop a CMA-focused guidance document, later published as Milk Allergy in Primary care (MAP) guideline, along with other infant feeding issues. MAP was designed for shortcomings of national and international guidelines in the initial clinical recognition and management of mild to moderate non-IgE-mediated disease in primary care.

## Methods

Longitudinal data collected of infant feeding products prescribed by general practitioners (GP's) in NI from June 2012 to November 2013 were analysed to assess variations in prescription patterns. Intervention of the Health and Social Care Board (HSCB) in February 2013 provided regional guidance with subsequent training of practical algorithms.

## Results

The total number of amino acid-based and extensively hydrolysed formulae significantly increased comparing a 6-month period from June to November in 2012 and 2013 before and after implementation (p<.05). The total quantity and total cost of colic treatments, lactose free formulae and comfort milks significantly decreased (p<.05). Over the study period, the percentage of recommended CMA prescribing increased from 12,9% to 24%, over 15,3% during the implementation period from October 2012 to February 2013 (p<.001 for trend).

## Conclusions

We present the first study evaluating the impact of CMA guidelines in the UK primary care. HSCB and MAP algorithms significantly increased the prescribed number of more expensive CMA formulae, at the cost of symptomatic colic, comfort and lactose free milks. The total cost of infant feeding products was however not affected. Practical advice and teaching of community-based health professionals allowed for effective implementation of regional guidelines, with appropriate diagnosis and treatment of a range of infant feeding issues and significant impact on GP prescription patterns. This shows promising results for randomised national research.

